# Visualization Experimental Study on Silicon-Based Ultra-Thin Loop Heat Pipe Using Deionized Water as Working Fluid

**DOI:** 10.3390/mi12091080

**Published:** 2021-09-07

**Authors:** Wenzhe Song, Yanfeng Xu, Lihong Xue, Huajie Li, Chunsheng Guo

**Affiliations:** School of Mechanical, Electrical & Information Engineering, Shandong University, Weihai 264209, China; 201800800305@mail.sdu.edu.cn (W.S.); 201916564@mail.sdu.edu.cn (Y.X.); 202017437@mail.sdu.edu.cn (L.X.); 201800800220@mail.sdu.edu.cn (H.L.)

**Keywords:** loop heat pipe, deionized water, two-phase flow, visualization, heat transfer experiment

## Abstract

As a type of micro flat loop heat pipe, s-UTLHP (silicon-based ultra-thin loop heat pipe) is of great significance in the field of micro-scale heat dissipation. To prove the feasibility of s-UTLHP with high heat flux in a narrow space, it is necessary to study its heat transfer mechanism visually. In this paper, a structural design of s-UTLHP was proposed, and then, to realize the working fluid charging and visual experiment, an experimental system including a holding module, heating module, cooling module, data acquisition module, and vacuum chamber was proposed. Deionized water was selected as a working fluid in the experiment. The overall and micro phenomena of s-UTLHP during startup, as well as the evaporation and condensation phenomena of s-UTLHP during stable operation, were observed and analyzed. Finally, the failure phenomenon of s-UTLHP was analyzed, and several solutions were proposed. The observed phenomena and experimental conclusions can provide references for further related experimental research.

## 1. Introduction

With the development of electronic technology, as the function of the chip becomes more and more powerful, its power consumption is also rises. At the same time, electronic products are developing in the direction of small, light, and thin, which has caused the heat flux of electronic components to rise sharply. Therefore, the evolution of mobile terminals has led to more stringent requirements on the size of electronic cooling components [1]. Taking smartphones as an example, in recent years, the power consumption of system chips of smartphones has increased to 3–5 W, while the thickness of smartphones has been reduced to about 6 mm [2]. When the equipment runs at high power, it produces a great deal of heat. This heat accumulation leads to uneven temperature distribution, and the resulting thermal stress causes thermal deformation of internal electronic devices [3]. There is evidence indicating that the micro flat loop heat pipe can maximally reduce the temperature of the chip and improve the overall temperature uniformity of the chip. Furthermore, its required heat dissipation space is very small. Thus, the micro flat loop heat pipe appears to be an ideal solution for high-intensity heat dissipation of micro-scale components [4].

The heat pipe is characterized by high thermal conductivity, long service life, convenient maintenance, and compact and flexible structure. Recently years, research and experiments on heat pipes have attracted more attention [5]. The focus of prior studies mainly differ from the following four aspects: microchannel design, working fluid selection, heat pipe performance, and visualization. In terms of microchannel research, Lim J. [6] proposed a channel layout for flat plate micro heat pipe under local heating conditions that can effectively overcome the limitations of local heating conditions. For working fluid research, Kim J. [7] used ethanol, FC-72, HFE-7000, R-245fa, and R-134a as experimental working fluids to study the selection criteria of working fluids in micro heat pipes. In addition, Narayanasamy M. [8] mixed acetone, deionized water, and tetrahydrofuran fluids with graphene oxide nanoparticles by ultrasonication and prepared nanofluids as working fluids for micro heat pipes for lithium-ion batteries. In terms of performance research, Zhao Y. N. [9] deduced the heat transfer performance of a micro heat pipe array, the influence of inclination angle on heat transfer characteristics, and the pressure limit of the heat pipe structure by using ammonia as a working fluid. Chen G. [10] researched the effects of heat load, cooling water temperature, inclination angle, and other factors on the thermal response performance, temperature distribution, thermal resistance, and other heat transfer characteristics of ultra-thin flat heat pipes. Wang G. [11] concentrated on the effects of working fluid type, charging rate, and inclination angle on the performance of flat plate micro heat pipes through experiments. In terms of visualization research, Kamijima C. [12] studied the relationship between the thermal characteristics and the internal flow characteristics of the micro heat pipe with FC-72 as the working fluid by measuring the effective thermal conductivity, visualizing the flow pattern, and simulating heat transfer according to the flow pattern. The study by Kim Y. B. [13,14] investigated the thermal flow and rapid thermal oscillatory flow in an asymmetric micro pulsation heat exchanger utilizing FC-72 and ethanol as working fluids. It also clarified the optimal charging rate of the two working fluids, evaluated their thermal resistances, identified their key mechanisms of circulating motion, and observed two different flow modes (oscillatory eruption mode and circulating mode). Our research group performed an in-depth study of the characteristics of microchannel flow using numerical simulation [15]. On the basis of our previous research and the four types of research listed above, we conducted in-depth research focusing on visualization and expanded the literature on the design of microchannels and the performance of heat pipes.

Microchannel systems can significantly improve energy transfer efficiency and reduce the overall size of chips. In various technical fields, microchannel technology is one of the most promising areas for the development of equipment [16]. As a kind of phase change heat transfer, gas–liquid two-phase flow heat transfer has very high efficiency [17]. The microchannel is etched on a silicon substrate, and the heat transfer is carried out by gas–liquid two-phase flow, which can realize a flat loop heat pipe with small size and high efficiency. Compared with other working fluids, deionized water has stronger polarity and stronger affinity with silicon chips, which are suitable for silicon-based heat pipes. The phase transition process, flow process, and vapor–liquid distribution of the working fluid can be observed by visualization experiment [18]. Therefore, in this study, a micro ultra-thin loop heat pipe was designed with silicon as the mainboard material, deionized water as the working fluid, and capillary force provided by the microchannel array. The phase transition behavior and vapor–liquid interface of the heat pipe during startup and stable operation were visualized through experiments. The results have an important reference value for the theoretical analysis of the heat transfer mechanism of ultra-thin loop heat pipes.

## 2. Structure and Experimental Setup of s-UTLHP

### 2.1. Structure of s-UTLHP

**Figure 1:**
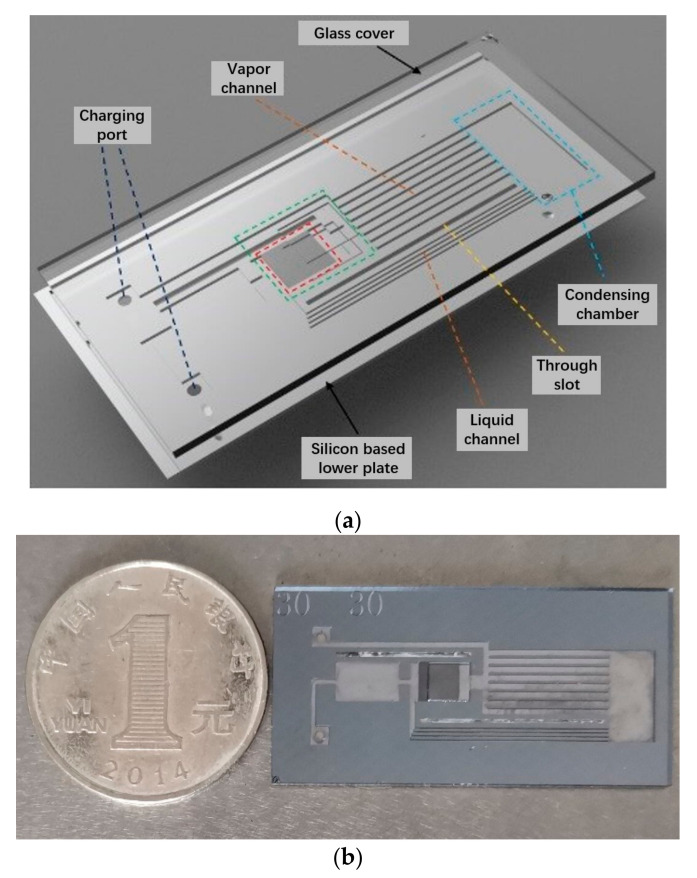
s-UTLHP prototype designed in this paper. (**a**) SolidWorks assembly sketch of s-UTLHP; (**b**) Physical drawing of s-UTLHP.

**Figure 2:**
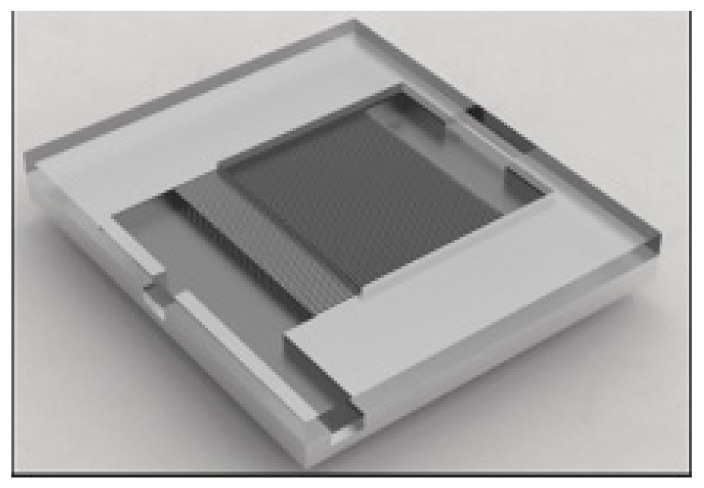
Structure diagram of the evaporator.

The main structure of the s-UTLPH designed in this study, as shown in Figure 1a,b, included a silicon-based motherboard and a Pyrex 7740 glass upper cover plate. The high boron glass upper cover plate was packaged with the silicon-based motherboard by bonding, which allowed us to observe the flow pattern’s evolution process in the microchannel visually and intuitively and to understand its physical mechanism. The silicon-based motherboard comprised an evaporation chamber, a condensation chamber, a vapor channel, and a liquid channel connecting the evaporation chamber and the condensation chamber. The overall s-UTLPH size was 40 mm × 20 mm × 1.45 mm. The capillary force of s-UTLPH was provided by parallel microchannels. Both the depth of the microchannel and the whole etching depth were 180 μm. The width of the microchannel was 30 μm, and the aspect ratio was 6:1. In addition, a rectangular vapor overflow cavity was etched on the corresponding glass cover plate above the parallel microchannel. The height of the vapor overflow cavity was 240 μm, and its structure is displayed in Figure 2.

The evaporation chamber converted the working fluid into vapor by heating in the evaporation chamber, and the capillary core inside provided the capillary driving force to ensure the unidirectional circulation of the working fluid. The main function of the condensing chamber was to condense the vapor working fluid back to the liquid working fluid to release heat. The compensation chamber can store the liquid working fluid of cooling backflow, which was convenient to supplement the liquid working fluid in time and prevent the “dry burning” of the cutoff flow. Eight vapor channels and four liquid channels were located between the evaporation chamber and the condensation chamber. The vapor working fluid produced by phase change flowed to the condensation chamber through the vapor channel. When the condensation chamber became cold, the working fluid turned into liquid and then flowed back to the evaporation chamber through the liquid channel. A hollow insulating slot was also arranged on the s-UTLHP to reduce heat leakage. The size of the s-UTLHP devised in this study is presented in Table 1.

### 2.2. Experimental Device

**Figure 3:**
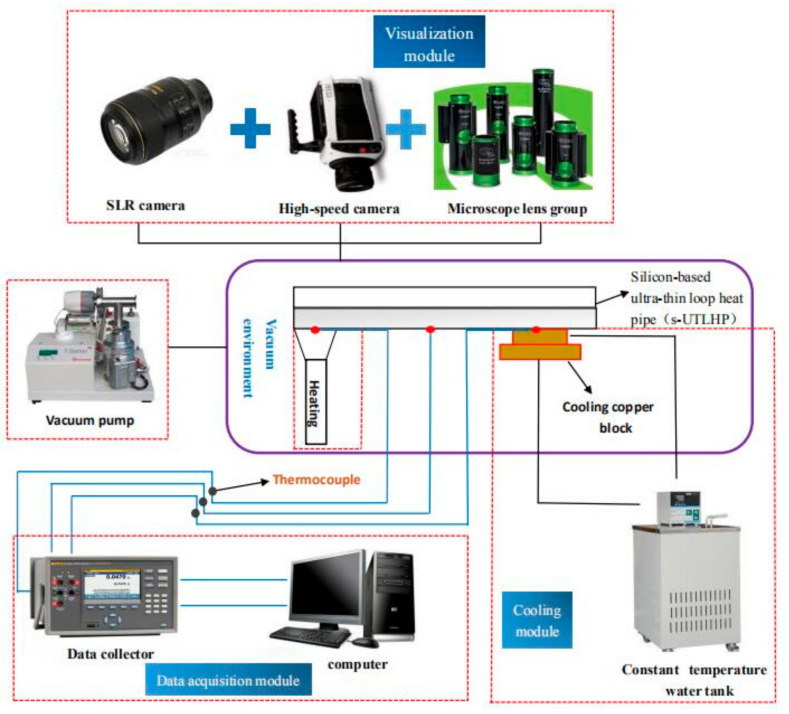
Visualization of the heat transfer experiment system.

To meet the requirements of the working fluid charging, the visualized heat transfer experiment, and the s-UTLHP measurement, it was necessary to arrange the experimental system around the s-UTLHP with limited space. As shown in Figure 3, the visualized heat transfer experimental system involved an s-UTLHP holding module, heating module, cooling module, data acquisition module, vacuum chamber, etc. The data acquisition module consisted of a temperature data acquisition module and a visualization module.

In this study, the silicon-based bottom plate of the s-UTLHP was set with two outlets for charging and pumping. First, a mechanical pump was used for primary air extraction, and an Edward molecular pump was utilized for secondary air extraction. When the pressure was below 1.5 × 10^−3^ Pa, the valve at the air extraction port was closed, and then the valve on the other side was opened to fill the working fluid. Deionized water was applied as the working fluid in this paper. The physical parameters of this working fluid are presented in Table 2. After vacuumizing the inner part of the s-UTLHP and its connecting pipelines, the charging operation of the s-UTLHP was carried out. The experimental results demonstrated that high vacuum degree and good charging effect could be obtained by the vacuum charging method, which provides a guarantee for the rapid startup and stable operation of s-UTLHP.

**Figure 4:**
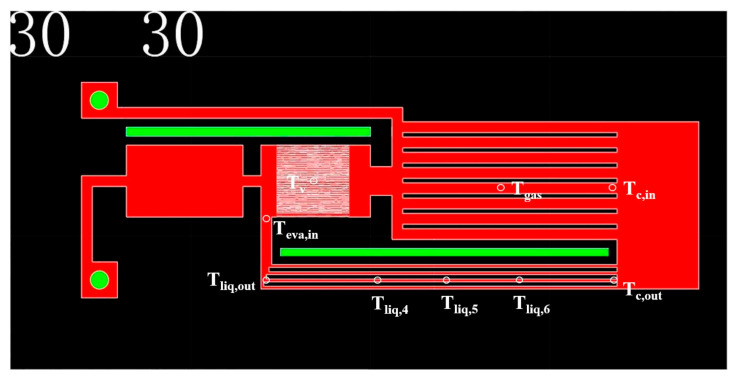
The layout of the thermocouple for temperature measurement. T_eva, in_—evaporation chamber inlet, T_liq, out_—liquid line outlet, T_liq,4,5,6_—liquid pipeline, T_c,out_—condensation chamber outlet, T_gas_—gas channel, T_v_—evaporation chamber, T_c,in_—condensation chamber inlet.

The s-UTLHP, after charging and sealing, was accurately heated by a ceramic heating plate combined with a heat transfer copper block. An ITECH programmable power supply was used of the model IT6121B. Furthermore, a copper cooling block was attached to the condensing end. Constant-temperature cooling water was continuously supplied by a constant-temperature circulating water cooler. The temperature of the condensing end was adjusted by controlling the temperature of the circulating water. In the experiment, the cooling water temperature was set to 5 °C. During the experiment, the Omega T thermocouple Was employed to detect the temperature of each key node, and Fluke2638A was used to record and store the temperature data. The arrangement of the thermocouple is displayed in Figure 4. The temperature measuring point was also set to detect the room temperature.

### 2.3. Error Analysis and Data Processing

#### 2.3.1. Error Analysis

In this experiment, contact and non-contact temperature measurement methods were adopted through a combination of thermocouple and infrared temperature measurement. In contact measurement, thermocouples were pasted on the s-UTLHP silicon substrate, and thermally conductive silicone grease was coated between the heating block and the silicon substrate to reduce the contact thermal resistance. Meanwhile, the PTFE fixture was designed to reduce the heat loss of the insulation section. The parameters or data to be measured included charge statistics, surface temperature measurement, and input heat calculation. Hence, the main experimental error sources were the temperature measurement point, charge mode, test error, and DC power supply instrument.

(1) Charge uncertainty

A Hamilton 250 μL injector was used to achieve quantitative charging. The minimum calibration interval was 2.5 μL, and the error was ±1.25 μL.

(2) Temperature uncertainty

To ensure the repeatability and effectiveness of the experimental phenomenon, the method of repeated testing was utilized for the experiment under the same conditions. The directly measured parameter value (temperature) in the experiment was calculated according to the following formula:

$$U\left(T_{i}\right)=\sqrt{\frac{1}{I\left(J-1\right)}\sum_{j=1}^{J}\sum_{i=1}^{I}(T_{ij}-\bar{T_{i}})}$$

In the above formula, *J* is the serial number of the repeated experiment and *i* is the serial number of the temperature point tested. In the test results, the maximum deviation of temperature measured by the thermocouple was 1.56 °C, and the corresponding maximum uncertainty was 1.93%.

(3) Uncertainty of DC power supply

The uncertainty caused by the instrument is given by class B uncertainty $U\;\left(E\right)=\frac{A}{k}$, where A is the nominal error in the instruction manual of the instrument and *k* = $\sqrt{3}$. In the experiment of s-UTLHP heat transfer characteristics, the input heat load was changed by adjusting the voltage of the DC regulated power supply. The nominal error was ±1%, and the standard uncertainty of voltage was $U\left(V\right)=1\%\sqrt{3}=0.577\%$. According to the principle of error transfer, the uncertainty of input heat load Q_in_ was $U\left(Q_{in}\right)=\frac{\sqrt{{\left(I\cdot u\left(V\right)\right)}^{2}+{\left(V\cdot u\left(I\right)\right)}^{2}}}{V\cdot I}=2.341\%$.

#### 2.3.2. Data Processing

The s-UTLHP charging rate is defined as the ratio of the volume of the working fluid to the total volume of the system. The specific value is computed by the following formula:

$$\Phi =\frac{V_{wf}}{V_{sys}}$$

where Φ is the charging rate, $V_{wf}$ is the volume filled with liquid working fluid, and $V_{sys}$ is the volume of the s-UTLHP system.

## 3. Analysis and Discussion of Experimental Results

### 3.1. Startup of the s-UTLHP

#### 3.1.1. Overall Phenomena at Startup

For the operation of traditional heat pipes, a suitable working fluid should have high latent heat of vaporization and surface tension, low viscosity, and good wetting performance. Additionally, a working fluid with different critical points should be selected to conform to the temperature range [19]. In this study, deionized water was selected as a working fluid.

**Figure 5:**
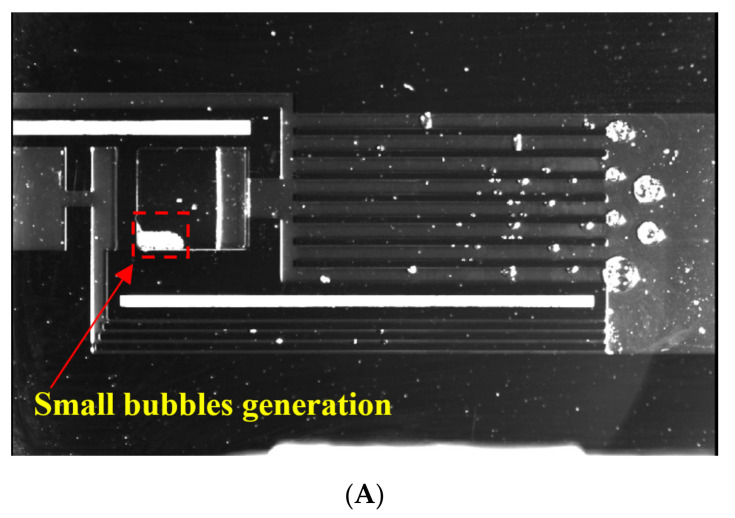

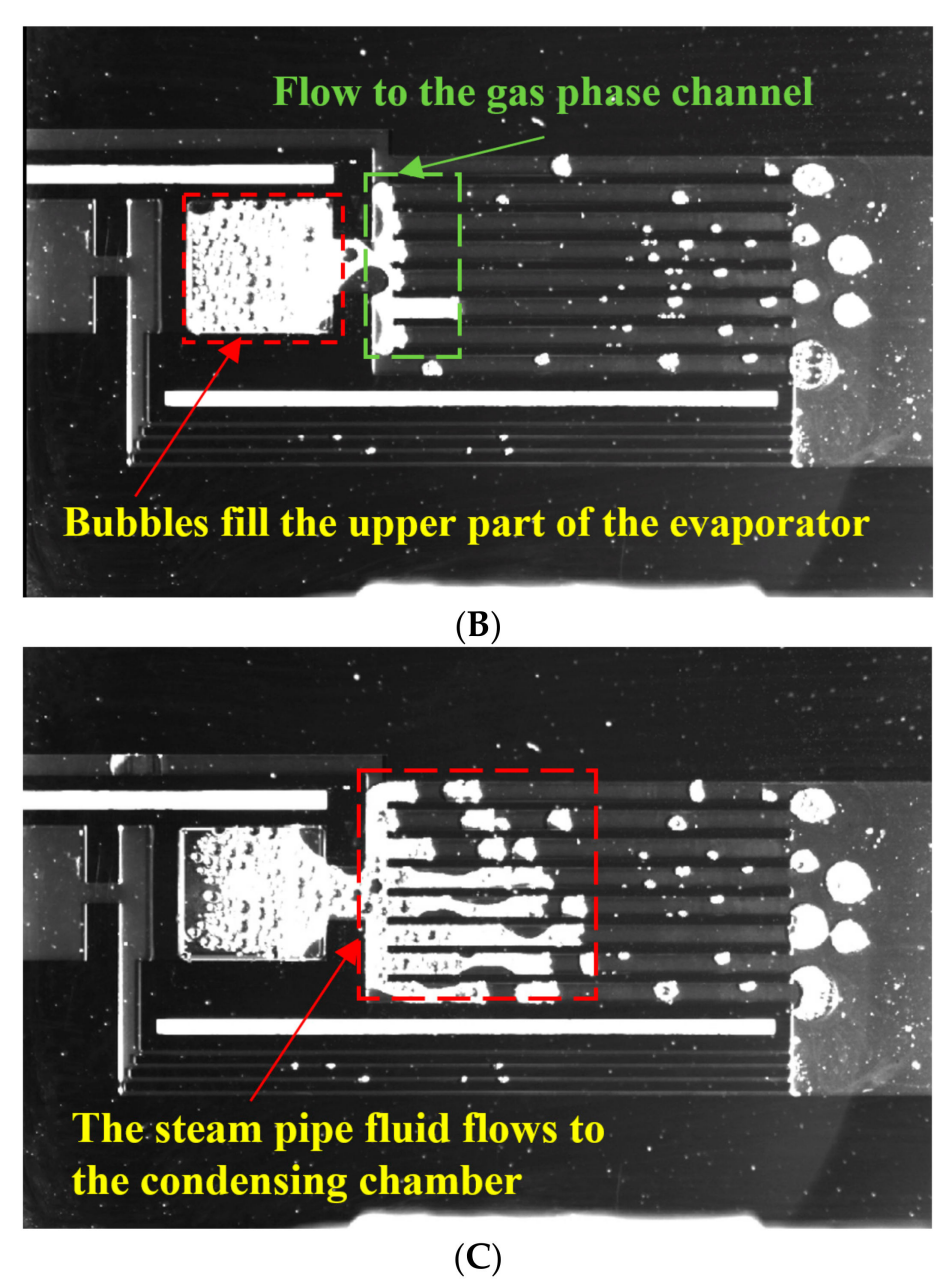
Startup process of the s-UTLHP with two different charging working fluids: (**A**) bubble generation stage at microchannel; (**B**) beginning stage of vapor outflow; (**C**) stable stage after vapor propulsion.

The startup process of the s-UTLHP filled with deionized water working fluid is presented in Figure 5. The voltage of the regulated power supply was set at 3.4 V, and the current change was recorded after stable operation. The heating power was 5.54 W, the effective heat transfer area was 4 mm × 3 mm, and the heat flux was about 46 W/cm^2^.

Small bubbles appeared when t = 5 s (Figure 5A). During the experiment, the liquid working fluid in the s-UTLHP evaporation chamber absorbed the external heat through the chip substrate and increased in temperature. After reaching the saturation temperature, nucleate boiling occurred in the parallel microchannel. The vapor bubbles in the vapor overflow cavity above the microchannel expanded after their appearance to occupy the whole vapor overflow cavity within 5 s (Figure 5B). With a continuous heating process or with an increase in heating power, the vapor bubbles above the vapor overflow cavity decreased and were replaced by pure working fluid vapor. The reason was that the vapor bubbles on the upper cover plate of the evaporation chamber were mixed with the surrounding droplets (liquid film), which was the final result of the dynamic process of evaporation and condensation in the evaporation chamber. Higher heat input made the evaporation effect far greater than the condensation effect, so that purer vapor appeared in the evaporation chamber. In the meantime, the working fluid vapor began to flow from the vapor overflow chamber to the vapor pipe, and the vapor continuously pushed the liquid in the vapor pipe to flow to the condensation chamber (Figure 5C). From Figure 5B,C, it can be seen that the continuous generation of vapor bubbles in the evaporation chamber was weakened, the nucleate boiling was replaced by thin-film evaporation, and the liquid pipe and compensation chamber were filled with liquid and continuously replenished the evaporation chamber. At this time, the s-UTLHP started up and ran stably. In the initial stage of heating and evaporation, it took only 14 s for the deionized water to advance to the vapor channel from the generation of bubbles to the evaporation drying of the vapor overflow chamber.

At the primary stage of startup, the area above the parallel microchannel array of the evaporation chamber evaporated first. During the phase transition of the deionized water working fluid, numerous bubbles were generated, and then a large number of fine droplets were formed, as displayed in Figure 5B,C. Moreover, nucleate boiling emerged in the parallel microchannel at the initial stage of operation.

#### 3.1.2. Micro Phenomena during Startup

**Figure 6:**
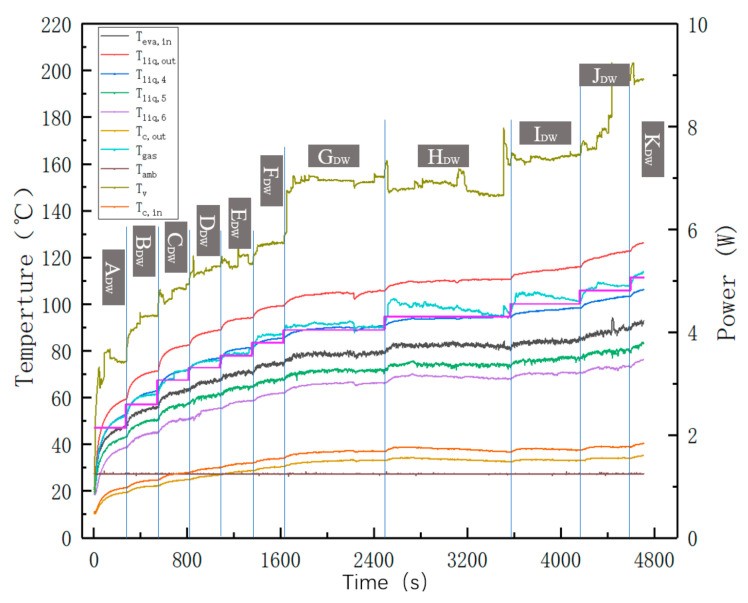
Relationship among the temperature and power of s-UTLHP and the time. T_eva,in_ is the inlet temperature of the evaporation chamber; T_liq,out_ is the outlet temperature of the liquid pipeline; T_liq,4,5,6_ is the temperature of the liquid pipeline, evenly arranged along the liquid return direction; T_c,out_ is the outlet temperature of condensation chamber; T_gas_ is the temperature of the vapor phase pipeline; T_amb_ is the ambient temperature; T_v_ is the temperature of the evaporation chamber; and T_c,in_ is the inlet temperature of the condensation chamber.

To have a more comprehensive understanding of the internal startup and evaporation boiling phenomena for the s-UTLHP, the microscopic phenomena of the s-UTLHP under variable power were observed, and the temperature data during the experiment were recorded. Deionized water has the advantages of being non-toxic and easy to obtain and of having a high latent heat of vaporization, which is commonly used as a working fluid for loop heat pipes [20]. The startup and variable power loading experiments for the s-UTLHP were carried out with deionized water as the working fluid, and the charging rate was 90%. In the experiment, we increased the input power at equal intervals. After each increase of the input power, different heat transfer phenomena occurred. According to these phenomena, we divided the stage into different regions. In addition, each time we heating at the regular rate is a variable power test after reaching steady state at a specific power. The sign of reaching steady state is that the temperature changes very little (gradually flattening from the temperature curve, that is, the temperature is stable or fluctuates within a certain range with the passage of time at a certain power). In this process, the relationship between the overall temperature and power within the experimental time is shown in Figure 6. Ten variable power loading experiments were carried out on the s-UTLHP, and the resulting curve was divided into 11 regions according to the variable power loading time. It can be seen from the figure that the s-UTLHP could respond quickly to different power and heat loads. For the evaporation chamber outlet temperature curve (the highest temperature curve in the figure), after changing the heating power, the temperature first rose rapidly, then gradually decreased and stabilized, and then fluctuated within a certain range. This was because the fluctuation of vapor flow resulted in the fluctuation of heat carried away, which gave rise to the fluctuation of temperature. The temperature of the liquid phase pipeline gradually increased along the fluid return path, because the position near the end of the return pipeline was close to the heating source, which could conduct part of the heat.

**Figure 7:**
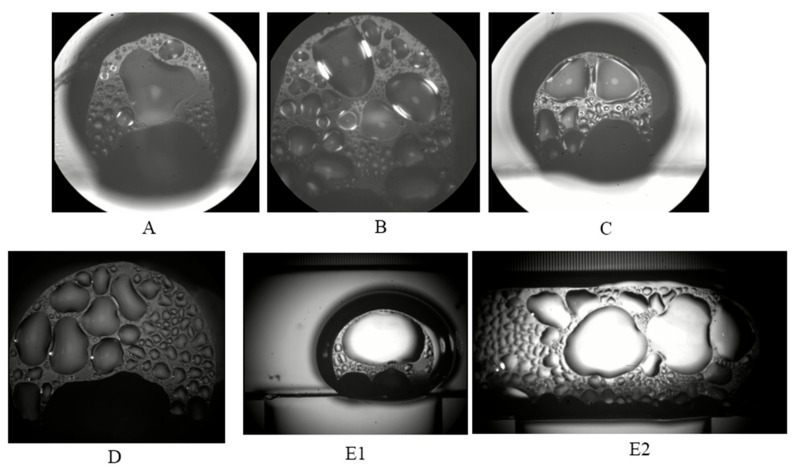
Startup process of the s-UTLHP.

According to the above two startup judgment marks for the s-UTLHP, the startup power of the s-UTLHP with deionized water as the working fluid was 3.5517 W, which corresponds to phase E in the curve. The temperature difference between the inlet and outlet of the condensation chamber was 3.18 °C. The whole change process of the bubbles in the evaporation chamber during startup (shown in Figure 7) occurred in the area without the shallow cavity and microchannel in the evaporator. The change of bubble volume in stages A–D was not obvious and was accompanied by expansion–contraction oscillation with smaller volume change amplitude, but the magnification was different across the four stages. There were also both large and small droplets, which were constantly generated, merged, grown, and disappeared. The disappearance here was integration either with the liquid around the bubble or with the liquid below. At the E stage, the bubble volume increased rapidly and almost covered the whole area without the shallow cavity and microchannel in the evaporator.

### 3.2. Stable Operation of the s-UTLHP

#### 3.2.1. Evaporation Phenomena in Stable Operation

The steady-state performance of the heat pipe was reflected in the variable power operation experiment to a certain extent [21], and the micro phenomena of vapor–liquid distribution, evaporation, and condensation were also captured. In the variable power operation of s-UTLHP, it was found that, as shown in Figure 6, when the power increased, the temperature responded in time, showing a ladder shape on the whole. The temperature curves of the single stages showed a trend of first increasing and then becoming basically stable.

**Figure 8:**
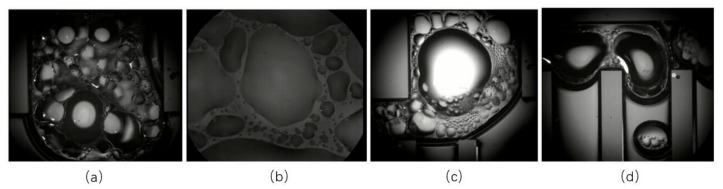
Microscopic images of the s-UTLHP at different stages of steady-state operation: (**a**) Image of the connection between the evaporator and the vapor phase line; (**b**) Image inside the evaporator; (**c**,**d**) The image of the inlet of the vapor phase pipeline during stable operation.

As presented in Figure 8, when deionized water was applied as a working fluid, the s-UTLHP operated stably from stage F. In the subsequent stages, the inlet temperature curve of the vapor phase pipeline was generally stepped, and for each stage, it first increased, then decreased, and then generally stabilized. This was because, for the ceramic heating plate under a given voltage, over time, the current gradually decreased and then stabilized. The microscopic image of stage F at the junction of the evaporation chamber and vapor phase pipeline is presented in Figure 8a. Many droplets can be seen; these droplets constantly repeated the process of generation, growth, disappearance, and washing away. As the power increased, the process changed faster and faster. Figure 8b shows the phenomena in the evaporation chamber. Figure 8c,d show the bubbles at the inlet of the vapor phase pipeline during the stable operation time. When the deionized water working fluid shrank and broke at the inlet of the vapor phase pipeline, slug-like flow appeared, and adjacent bubbles did not easily fuse.

#### 3.2.2. Condensation Phenomena in Stable Operation

**Figure 9:**
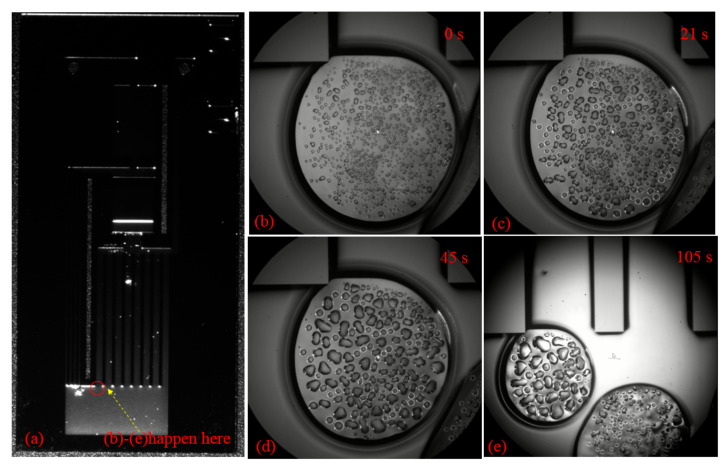
Bubble condensation process in the condensation chamber: (**a**) illustrates where (**b**–**e**) occur in the condenser; (**b**–**e**) are the images at 0 s, 21 s, 45 s and 105 s respectively.

Like an ordinary loop heat pipe, the s-UTLHP transfers heat through the evaporation–condensation phase transition of working fluid. Figure 9 displays the bubble condensation process in the condensation chamber during stable operation. The positions of Figure 9b–d are illustrated in Figure 9a. These images were obtained under the same magnification and field of view to observe and compare the change of bubble volume. Taking the NCG bubble in focus clearly within the field of view as the initial timing, the 0 s bubble phenomenon is shown in Figure 9b. The condensation process occurred when the heat flux was 37.5575 W/cm^2^.

There were many obvious droplets in the deionized water working fluid bubbles. At first, the droplets were small, round, separated from each other, and independent in shape, as shown in Figure 9b. When the edges of the two bubbles grew together as the condensation continued, the condensable components in the bubble gradually condensed. As a result, the droplets were produced or merged with the existing droplets, leading to an increase in the droplet density in the bubble. As shown in Figure 9c,d, the edges of the two bubbles tended to gradually separate. The reason is that the volume of the bubble decreased and the volume of the droplet inside the bubble became larger than depicted in Figure 9c. The two bubbles were completely separated at the time of Figure 9e. The bubble edge of the object was visible, the bubble volume was significantly smaller, and the internal droplet volume became larger.

**Figure 10:**
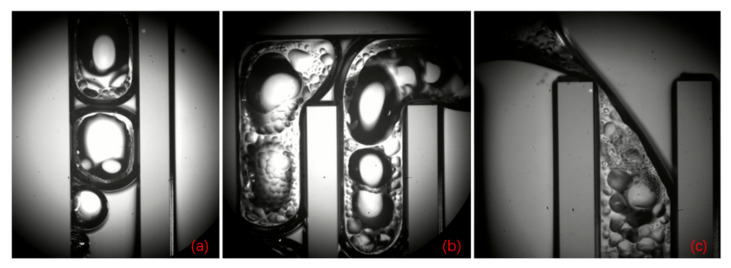
Condensation process in vapor phase pipeline.

It was observed that the condensation phenomenon of this s-UTLHP could occur in the gas-phase pipeline when the heat flux was moderate. Bubbles of deionized water working fluid in the gas-phase pipeline during stable operation are shown in Figure 10a–c. The deionized water was ejected into independent bubbles in the gas-phase pipeline, and it was difficult for those bubbles to fuse, even if they shared part of a bubble boundary, as shown in Figure 10a. This was related to the higher surface tension and lower viscosity of deionized water. The volumes of the four bubbles displayed in Figure 10a were different. The lower one had experienced a long time of condensation in the vapor line, while the upper one had just left the column. In the condensation process, slug flow occurred; that is, the vapor column broke at a certain distance, forming independently isolated bubbles. As described above for the condensation chamber, the bubbles were not always completely condensed, and the condensation rate was slow. During operation, the separating vapor column was positioned at the entrance of the rightmost vapor phase pipeline, as shown in Figure 10c, because of the stress at the corner.

### 3.3. Failure of s-UTLHP

**Figure 11:**
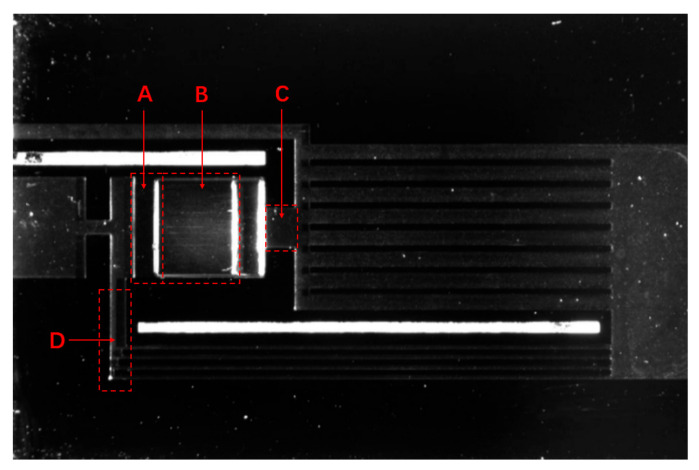
s-UTLHP structure diagram.

When the heat flux continued to increase, the equilibrium between the refrigerant reflux and phase transition overflow was broken, and the capillary core was considered to be burnt out [22]. Compared with capillary cores made of porous media, the design of a large number of high-aspect-ratio microchannel arrays can make a system have higher stability, effectively reduce the flow resistance, improve the permeability of the evaporation chamber, ensure that the liquid can flow back in time under high heat flux and have higher mass flow rate, and delay the occurrence of flow interruption and drying phenomenon [23]. In this design, the compensation chamber compensated the backflow of liquid in time before the system started up and when the heat flow changed rapidly, so as to improve the stability of the continuous operation of the s-UTLHP. Furthermore, the high-aspect-ratio microchannel array was equipped with a wide pure microchannel area (as shown in area A in Figure 11). This area could generate a great capillary pressure difference through the microchannel under the condition of high heat flux and gas–liquid two-phase transition, thus forming a “hydraulic lock” and effectively preventing backflow. In area B, there was also a microchannel array with a high aspect ratio on the silicon substrate, but unlike in area A, there was a vapor overflow cavity above area B. Figure 11, area C shows the outlet of the vapor overflowing from the evaporation chamber to the gas-phase pipeline, and area D shows the inlet of the liquid working fluid reflux. By setting a reasonable proportion, the circulating pressure change of the working fluid in the s-UTLHP could be effectively balanced.

**Figure 12:**
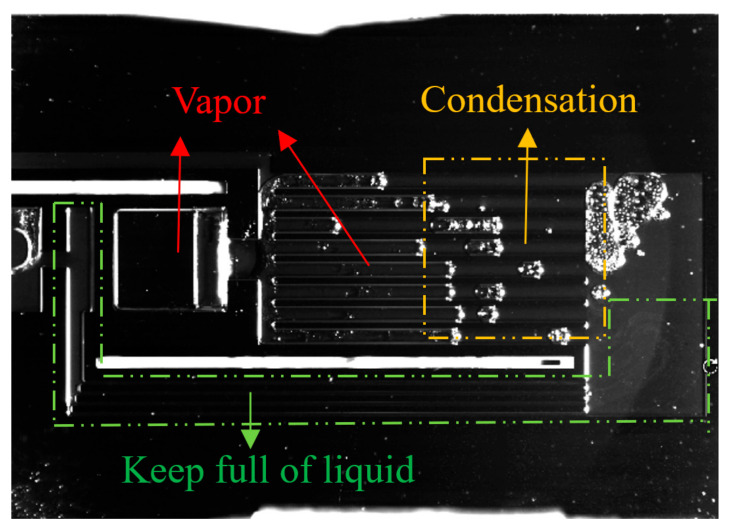
Imaging of the s-UTLHP under high heat flux.

An image of the s-UTLHP filled with deionized water working fluid under high heat flux is shown in Figure 12. When the image was taken, the heat input was 41.3 W/cm^2^. It can be observed that under relatively high heat flux, the gas phase pipeline appeared foggy because of two-phase flow. This phenomenon occurred because a great number of gaseous working fluids produced by phase transformation carried small droplets when flowing, and the pressure in the pipeline was high. Furthermore, there was liquid film attached to the wall of the gas phase pipeline. The vapor phase working fluid was produced by continuous phase change in the evaporation chamber. The vapor phase working fluid continuously advanced to occupy most of the gas phase pipeline and was in a relatively stable state.

**Figure 13:**
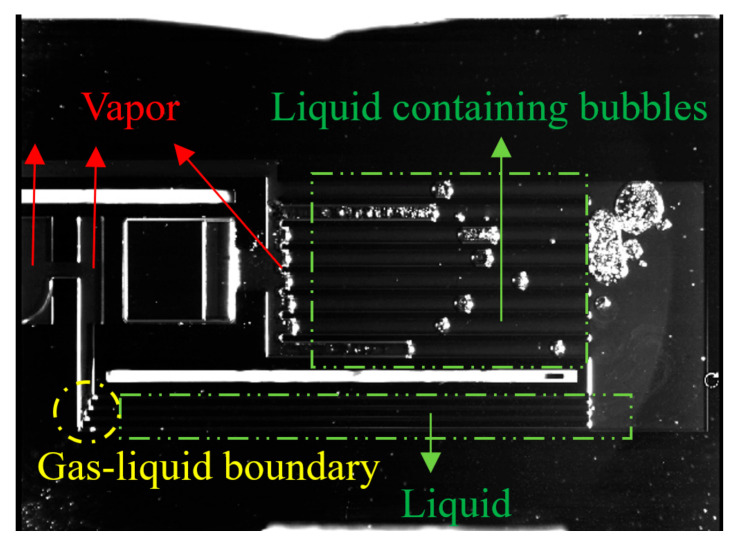
Imaging of s-UTLHP during drying.

The s-UTLHP in this study had a certain operating limit. When the heat flux reached a certain upper limit, a “dry burning” phenomenon appeared in the evaporation chamber. “Dry burning” refers to the state wherein the working fluid at the inlet and outlet of the evaporation chamber begins to evaporate while the evaporation chamber is continuously heated, finally resulting in the vaporization of all the liquid working fluid in the evaporation chamber. This occurs because the liquid replenishment rate is less than the phase change rate of the working fluid, which means that the liquid cannot be replenished, the liquid film on the wall of the silicon-based microchannel is evaporated quickly, and the gas–liquid two-phase interface regresses until the liquid working fluid in the evaporation chamber completely changes phase. At this time, the single gas-phase state does not have the conditions for capillary force generation and cannot be recycled. As presented in Figure 13, the s-UTLHP filled with deionized water was dried at a heat flux of 44.8 W/cm^2^. Another reason for the drying is that the pressure of the non-condensable gas bubble itself increased due to continuous heating and combined with the vapor. The gas phase flowed back to the evaporation chamber and then dried up instantly; after that, the gas–liquid interface at the gas phase pipeline retrogressed. Because the capillary limit of s-UTLHP was not been reached, the heat flux could further improved if the influence of non-condensable gas was excluded.

In summary, by analyzing the operation state of the s-UTLHP filled with deionized water, we concluded that it was suitable for heat dissipation with high heat flux and environments that are sensitive to temperature fluctuations. In addition, the s-UTLHP failed because of insufficient liquid refrigerant supply after reaching the limit heat flux and because of non-condensable gas reflux.

## 4. Conclusions

In this study, an s-UTLHP with a size of 40 mm in length, 20 mm in width, and 1.5 mm in thickness was designed. The width of the parallel microchannel was 30 μm, and the aspect ratio was 6:1. The design could achieve high capillary force and high mass flow. Furthermore, the longitudinal section of the microchannel with a high aspect ratio could fully extend the liquid film and realize heat exchange with heat flux of 37.5575 W/cm^2^.

Deionized water was selected as the working fluid. In the charging experiment, the s-UTLHP was pumped and charged, and the non-condensable gas was eliminated by combining with visualization. After the s-UTLHP was separately charged with deionized water, it could run stably for a long time. When the heat flux exceeded the limit, the “dry burning” phenomenon was observed, which caused the failure of the s-UTLHP. At low heat flux, the evaporation chamber presented the coexistence of bubbles and droplets, that is, the dynamic equilibrium of evaporation and condensation. At high heat flux, there was pure gas in the evaporation chamber, and the evaporation rate was much higher than the condensation rate, so an efficient evaporation replenishment mechanism was carried out.

This study explored a self-circulating heat dissipation scheme suitable for microchip integration, realized phase-change heat transfer on the surface of a silicon substrate, and proved the feasibility of the scheme for high-heat-flux heat dissipation in a narrow space. Moreover, the regular structure of the high-aspect-ratio microchannel array in the core of the evaporation chamber means that these arrays can be mass-produced by a 3D IC manufacturing process, which could help with popularization and application. In addition, this research adopted a visual method to conduct macroscopic and microscopic observational studies of evaporation and condensation, respectively, and to further explore the boiling flow and condensation cycle characteristics of the working fluid in the microchannel. It also provided references for the improvement of fine structure and surface morphology and for the selection of a working fluid, which can enhance the heat transfer capacity and the stability of the cyclic heat transfer mechanism. Therefore, this study has important reference value in the field of micro-scale heat dissipation for electronic chips.

## Figures and Tables

**Table 1:** s-UTLHP dimension parameters.

s-UTLHP Components	Area (mm^2^)
Evaporation chamber	24
Liquid storage chamber	26
Condensing chamber	42.315
Vapor channel	57.6
Liquid channel	11.73

**Table 2:** Parameters of each liquid working fluid at standard atmospheric pressure and room temperature.

Fluid Working Fluid	Melting Point (°C)	Boiling Point (°C)	Surface Tension Coefficient (n/M)	Viscosity Coefficient (mPa*s)	Belgium KJ/(kg*K)
H_2_O	0	100	0.075	0.083	4.18

## Data Availability

The data that support the findings of this study are available from the authors upon reasonable request. The data are not publicly available due to that they are planned to be used in our following experiments.

## Nomenclature

*T*	temperature, °C
*U*	voltage, V
*I*	current, A
*J*	serial number of the repeated experiment
*i*	serial number of the temperature point tested
*A*	nominal error
V_wf_	volume filled with liquid working fluid, m^3^
V_sys_	volume of the s-UTLHP system, m^3^
*t*	time, s
Greek symbols	
Φ	charging rate
